# The NExT trial: Protocol for a two-phase randomized controlled trial testing transcranial magnetic stimulation to augment exposure therapy for youth with OCD

**DOI:** 10.1186/s13063-024-08629-1

**Published:** 2024-12-18

**Authors:** Christine Conelea, Claire Breitenfeldt, Alixandra Wilens, Linda Carpenter, Benjamin Greenberg, Jennifer Herren, Suma Jacob, Charles Lewis, Nicole McLaughlin, Bryon A. Mueller, Steve Nelson, Erin O’Connor, Giulia Righi, Alik S. Widge, Mark Fiecas, Kristen Benito

**Affiliations:** 1 Department of Psychiatry and Behavioral Sciences, University of Minnesota, Minneapolis, MN USA; 2 Pediatric Anxiety Research Center at Bradley Hospital, East Providence, RI USA; 3 COBRE Center for Neuromodulation, Butler Hospital, Providence, RI USA; 4 Department of Psychiatry and Human Behavior, The Warren Alpert Medical School of Brown University, Providence, RI USA; 5 Division of Biostatistics and Health Data Science, University of Minnesota, Minneapolis, MN USA; 6 Center for Neurorestoration and Neurotechnology, VA Providence Healthcare System, Providence, RI USA; 7 Department of Psychiatry and Biobehavioral Sciences, Semel Institute for Neuroscience and Human Behaviors, UCLA, Los Angeles, CA USA; 8 Department of Pediatrics, University of Minnesota, Minneapolis, MN USA; 9 Masonic Institute for the Developing Brain, University of Minnesota, Minneapolis, MN USA

**Keywords:** Transcranial magnetic stimulation, Neuromodulation, Cognitive Behavioral Therapy, OCD, Exposure therapy

## Abstract

**Background:**

Exposure with Response Prevention (ERP) is a first-line treatment for OCD, but even when combined with first-line medications it is insufficiently effective for approximately half of patients. Compulsivity in OCD is thought to arise from an imbalance of two distinct neural circuits associated with specific subregions of striatum. Targeted modulation of these circuits via key cortical nodes (dorsolateral prefrontal cortex [dlPFC] or presupplementary motor area [pSMA]) has the potential to improve ERP efficacy by decreasing compulsions during therapy.

**Methods:**

The NExT (Neuromodulation + Exposure Therapy) trial is a two-phase, multisite early-stage randomized controlled trial designed to examine whether TMS augmentation of ERP alters activity in dlPFC and/or pSMA-associated circuitry and reduces compulsions during therapy in youth with OCD age 12–21 years. Phase 1 (*N* = 60) will compare two different active TMS regimens with sham: A. continuous theta burst stimulation (cTBS) to pSMA vs. B. intermittent theta burst stimulation (iTBS) to dlPFC. A priori “Go/No-Go” criteria will inform a decision to proceed to Phase 2 and the choice of TMS regimen. Phase 2 (*N* = 60) will compare the selected TMS regimen vs. sham in a new sample.

**Discussion:**

This trial is the first to test TMS augmentation of ERP in youth with OCD. Results will inform the potential of TMS to enhance ERP efficacy and enhance knowledge about mechanisms of change.

**Trial registration:**

ClinicalTrials.gov NCT05931913. Registered prospectively on July 5, 2023.

## Administrative information

Note: the numbers in curly brackets in this protocol refer to SPIRIT checklist item numbers. The order of the items has been modified to group similar items (see http://www.equator-network.org/reporting-guidelines/spirit-2013-statement-defining-standard-protocol-items-for-clinical-trials/).

**Table:** 

Title {1}	The NExT trial: Protocol for a two-phase randomized controlled trial testing transcranial magnetic stimulation to augment exposure therapy for youth with OCD
Trial registration {2a and 2b}.	ClinicalTrials.gov Identifier: NCT05931913
Protocol version {3}	September 20, 2023 Protocol version 1
Funding {4}	This research is supported by the US National Institutes of Health (1R61MH133666-01, PIs: Benito & Conelea; 5P20GM130452).
Author details {5a}	Christine Conelea, PhD, Department of Psychiatry and Behavioral Sciences, Masonic Institute for the Developing Brain, University of Minnesota, Minneapolis, MN Claire Breitenfeldt, Department of Psychiatry and Behavioral Sciences, Masonic Institute for the Developing Brain, University of Minnesota, Minneapolis, MN Alixandra Wilens, Pediatric Anxiety Research Center at Bradley Hospital, East Providence, RI; The Warren Alpert Medical School of Brown University, Providence, RI Linda Carpenter, MD, Department of Psychiatry and Human Behavior, The Warren Alpert Medical School of Brown University, Providence, RI Mark Fiecas, PhD, Division of Biostatistics and Health Data Science, University of Minnesota, Minneapolis, MN Benjamin D. Greenberg, MD, PhD, Department of Psychiatry and Human Behavior, The Warren Alpert Medical School of Brown University Center for Neurorestoration and Neurotechnology, Providence, RI Jennifer Herren, PhD, Pediatric Anxiety Research Center at Bradley Hospital, East Providence, RI; The Warren Alpert Medical School of Brown University, Providence, RI Suma Jacob, MD, PhD, Adjunct Faculty at University of Minnesota; Division Director at Department of Psychiatry and Biobehavioral Sciences, Semel Institute for Neuroscience and Human Behaviors, UCLA, Los Angeles, CA Charles P. Lewis, MD, Department of Psychiatry and Behavioral Sciences, Masonic Institute for the Developing Brain, University of Minnesota, Minneapolis, MN Nicole McLaughlin, PhD, Department of Psychiatry and Human Behavior, The Warren Alpert Medical School of Brown University, Providence RI Bryon A. Mueller, PhD, Department of Psychiatry and Behavioral Sciences, University of Minnesota, Minneapolis, MN Steven M. Nelson, PhD, Department of Pediatrics, University of Minnesota, Minneapolis, MN; Masonic Institute for the Developing Brain, University of Minnesota, Minneapolis, MN Erin O’Connor, PhD, Pediatric Anxiety Research Center at Bradley Hospital, East Providence, RI; The Warren Alpert Medical School of Brown University, Providence, RI Giulia Righi, PhD, Pediatric Anxiety Research Center at Bradley Hospital, East Providence, RI; The Warren Alpert Medical School of Brown University, Providence, RI Alik Widge, MD, PhD, Department of Psychiatry and Behavioral Sciences, University of Minnesota, Minneapolis, MN Kristen Benito, PhD, Pediatric Anxiety Research Center at Bradley Hospital, East Providence, RI; The Warren Alpert Medical School of Brown University, Providence, RI
Name and contact information for the trial sponsor {5b}	K. Benito (principal investigator) Pediatric Anxiety Research Center (PARC), Bradley Hospital 1011 Veterans Memorial Pkwy East Providence, RI 02915 C. Conelea (principal investigator) Masonic Institute for the Developing Brain 2025 E. River Pkwy, Minneapolis, MN 55414
Role of sponsor {5c}	This is an investigator-initiated clinical trial. The funders played no role in the design of the study; collection, management analysis, and interpretation of the data; and writing of the report.

## Introduction

### Background and rationale {6a}

Obsessive–compulsive disorder (OCD) is a serious mental illness characterized by obsessions (intrusive thoughts or images that cause intense distress) and compulsions (actions designed to neutralize obsession-related distress). OCD affects 2–3% of youth and can cause significant impairments in social, academic, and familial functioning [1]. Symptoms remain chronic without treatment [2]. Exposure therapy (in the form of exposure plus response prevention; ERP) is an efficacious first-line therapy alone or in combination with serotonin reuptake inhibitors (SRI) for pediatric OCD [3]. However, many youth remain symptomatic following treatment, with only 53.6% achieving clinical remission in combined treatment, 39.3% in ERP alone, and 21.4–30.0% in SRI alone [3, 4]. There is a pressing need for augmentation strategies to improve these outcomes, and neuromodulation in particular has been identified as a highly promising method for ERP augmentation [5].

In ERP, youth engage in *exposures*—tasks that involve intentionally approaching obsession-related stimuli that elicit distress (e.g., touching an object perceived as contaminated). During exposures, *response prevention*—the prevention of related compulsions (e.g., hand washing)—is a necessary condition for fear extinction learning, the presumed mechanism of ERP, to occur. Use of strategies to reduce compulsions during exposure tasks predicts better outcomes at post-treatment and 3-month follow-up [6]. Unfortunately, exposure tasks are designed to elicit the urge to engage in compulsions, and resisting this urge can be very difficult for many youth. Improving youth’s ability to prevent compulsions during exposure tasks is a promising approach through which clinical response to ERP might be augmented.

Compulsive behavior is driven by aberrant functioning of cortico-striatal circuits involved in goal-directed vs. habitual action selection [7]. Goal-directed behaviors, which are contingency-sensitive and performed intentionally, depend on dorsomedial striatum (DMS, aka caudate) and its connections to dorsolateral prefrontal cortex (dlPFC). This circuitry shows *diminished* function in OCD, including within-and between-network resting-state hypoconnectivity [8] and local dlPFC hypoactivation during goal-directed planning tasks [9] and symptom provocation [10]. Habitual behaviors are repetitive, sequential behaviors that are contingency-insensitive, and these rely on connections between dorsolateral striatum (DLS, aka putamen) and sensorimotor cortices. This circuitry shows *exaggerated* function in OCD, including hyperactivity of the pre-supplementary motor area (pSMA), orbitofrontal cortex (OFC), DLS, and the pSMA-DLS circuit at rest, during response inhibition, and during symptom provocation (e.g., [10–12]. Collectively, these neural findings converge with behavioral evidence showing overreliance on the habit system relative to the goal-directed system in OCD [13].

Cortical nodes of these circuits (dlPFC, pSMA) have been leading candidates for studies using repetitive transcranial magnetic stimulation (rTMS) to target compulsivity. rTMS is a non-invasive brain stimulation technique that can selectively target cortical nodes of brain circuits in presumed “excitatory” (5 + Hz rTMS or intermittent theta bursting pattern, iTBS) or “inhibitory” directions (1 Hz rTMS or continuous theta bursting pattern, cTBS). rTMS modulates both the cortical node and its connected subcortical structures, [14] making it a useful tool for engaging specific functional brain networks with high resolution. TMS has traditionally been applied as a monotherapy for psychiatric illness. However, a large body of literature confirms that stimulation effects highly depend on the state of the targeted circuitry, [15] leading for calls to improve TMS outcomes with “functional targeting” that combines TMS with behavioral/cognitive engagement of the same circuit being modulated [16].

Enhanced function of DMS-driven circuitry is associated with ERP response in both adult [17] and pediatric [18] OCD. Excitatory stimulation with rTMS over cortical nodes has been linked to improvements in OCD symptoms globally (broad prefrontal cortex, dlPFC, though results are mixed [19]) and to compulsion reduction specifically (left dlPFC [20]). Reduction in the pathologically exaggerated function of DLS-driven habit circuitry is also linked to ERP response [21]. Inhibitory stimulation of pSMA with rTMS has been shown to reduce severity of OCD (e.g., [22]) and tics, [23] and inhibition of orbitofrontal cortex with cTBS reduces compulsive behavior [24].

A head-to-head examination of these rTMS strategies is needed to identify whether it is preferable to “turn up” goal circuitry or “turn down” habit circuitry to reduce compulsions during ERP. A meta-analysis concluded that both inhibitory pSMA stimulation and excitatory left dlPFC stimulation are efficacious for adult OCD, [25] but these have not yet been examined in the same study. The closest approximation is a retrospective chart review that found no difference in response rates for excitatory dlPFC and inhibitory pSMA stimulation [26].

Taken together, the existing literature suggests the hypothesis that augmenting ERP with rTMS to induce more normative/balanced function of circuits supporting goal-directed (dlPFC-DMS) and habitual behavior (pSMA-DLS) will boost response prevention, leading to enhanced ERP efficacy. Accordingly, we designed the NExT trial, an NIMH-funded two-phase, milestone-driven early-stage randomized controlled trial. To our knowledge, this will be the first treatment trial of rTMS for youth with OCD. Here, we describe the trial protocol.

### Objectives {7}

The overall objective of this two-phase study sequence is to test whether augmenting ERP with rTMS over cortical nodes of select cortico-striatal circuits changes circuit connectivity and enhances response prevention during exposure in youth with OCD. Phase 1 will test two rTMS strategies. In one, we will attempt to “turn down” overactive habit circuitry by delivering continuous theta burst stimulation (cTBS) to the pSMA. In the other, we will attempt to “turn up” underactive goal-directed circuitry by delivering intermittent theta burst stimulation (iTBS) to left dlPFC. Analyses will focus on testing changes in neural (fMRI-measured resting state functional connectivity; RSFC) and behavioral (rate of compulsions during ERP) outcomes.

We will proceed to Phase 2 if the following are demonstrated: (1) one or both active rTMS conditions demonstrate neural change (within- and between-subject effect size ≥ *d* = 0.3) in the targeted circuit post-treatment (pSMA-DLS for cTBS arm, dlPFC-DMS for iTBS arm); (2) evidence of safety (≤ 20% rate of TMS-related adverse events); and (3) evidence of feasibility (≥ 80% treatment completion rate). If these criteria are met, we will select a TMS regimen for phase 2 using the following rules: (1) if only one active condition meets the above criteria, that condition wins; (2) if both conditions meet criteria, the condition with lowest rate of compulsions during ERP wins.

The primary objective of Phase 2 is to replicate and validate Phase 1 findings for the optimal TMS regimen. Phase 2 will also test the link between neural/behavioral target engagement (change in RSFC and compulsions during ERP) and functional outcomes (OCD symptom change) at post-treatment. We will explore subgroup differences (e.g., based on OCD symptom subtype) and durability of treatment effects (at 1-month follow-up) in these primary outcomes. We will also explore the relationships between performance on computerized neurocognitive tasks with OCD symptom improvement.

### Trial design {8}

**Fig. 1:**
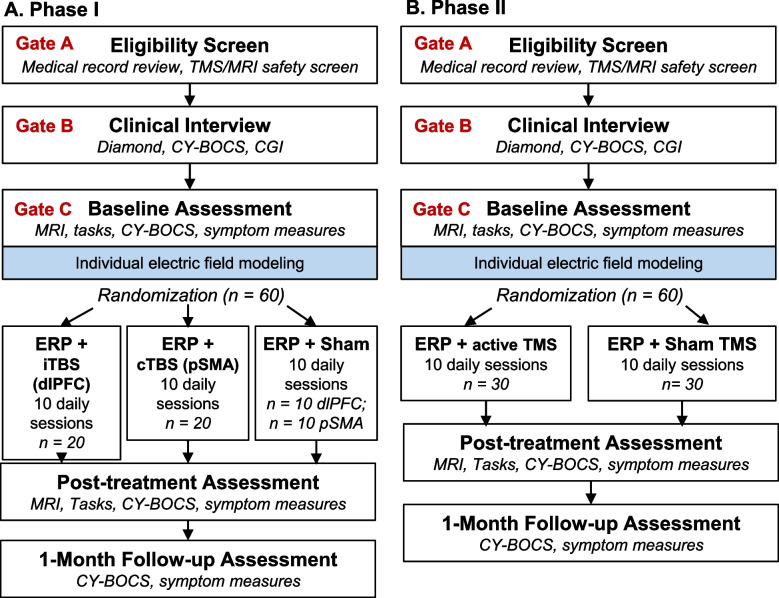
Flow chart of each study phase

Both phases are randomized, sham-controlled, double-masked, parallel-group, superiority trials. Phase 1 is a three-arm trial (sham, cTBS to pSMA, or iTBS to dlPFC; *n* = 20 per group), and phase 2 is a two-arm trial (sham or active stimulation; *n* = 30 per group; Fig. 1). Randomization in both phases will be blocked on site (Brown University or University of Minnesota) and symptom severity (Children’s or adult Yale-Brown Obsessive Compulsive Scale score above or below 32, indicating the cutoff for “extreme” symptoms). Participants have a 2 in 3 chance of being assigned to an active TMS treatment arm.

## Methods: participants, interventions and outcomes

### Study setting {9}

The study will occur in an outpatient clinical-research setting at two collaborating sites: the University of Minnesota (UMN) and Brown University (Brown). The “Brown” site encompasses two affiliated hospital sites in the Brown University Medical School system (Butler Hospital and Bradley Hospital).

## Eligibility criteria {10}

### Eligibility determination

Study staff will pre-screen interested participants over the phone for inclusion/exclusion criteria, including safety screening (Gate A). Relevant medical records will be reviewed by a study physician to assess initial eligibility. The clinical interview (Gate B) will include informed consent and a full clinical interview to determine final eligibility.

### Inclusion/exclusion criteria

The following are inclusion criteria: (1) age 12–21 years at the time of enrollment; (2) current OCD according to DSM-5 criteria; (3) score of ≥ 16 on the Children’s or adult Yale-Brown Obsessive Compulsive Scale (C/YBOCS), indicating OCD symptoms of moderate or greater severity; (4) presence of motor compulsions on CY-BOCS compulsion checklist; (5) participant and at least one parent (for participants < 18) are English-speaking, to ensure comprehension of informed consent and study measures and instructions; (6) medication stability for participants ≥ 6 weeks with no planned changes during the 3-week intervention protocol for those taking psychotropic medication.

The following are exclusion criteria: (1) personal or family history in a first-born relative of a medical condition judged by a study physician to impact the risk profile of TMS or participant’s ability to engage in the study; some examples include epilepsy or seizure disorder(s), bipolar disorder/mania, intracranial pathology, traumatic brain injury, brain tumor, stroke, implanted medical devices, or moderate-severe heart disease; (2) current pregnancy or youth of childbearing age not using effective contraception; (3) inability to undergo MRI; (4) left handedness; (6) active suicidality; (7) history or risk for neurocardiogenic syncope; (8) concurrent psychotherapy of any kind for OCD; (9) concurrent TMS or receipt of any TMS experimental or clinical treatment less than 3 months prior to enrollment; and (10) taking a medication deemed to pose high seizurogenic potential per physician review.

### Individuals who will perform the interventions

TMS operators will be trained according to recommended training guidelines [27]. ERP therapists will be individuals who have completed evidence-based training in the delivery of ERP for OCD across the lifespan [28] and are additionally trained in this study protocol. They will receive ongoing supervision from a licensed clinician with ERP expertise.

### Who will take informed consent? {26a}

Following Institutional Review Board (IRB) and Health Insurance Portability and Accountability Act (HIPAA) guidelines, trained research staff will obtain informed consent from parents (for minor participants age 12–17) or adult participants (age 18–21 years) and assent from minors. Informed consent will be obtained at the clinical interview (Gate B), prior to data collection. If any minors turn 18 during their participation, they will be re-consented as an adult.

### Additional consent provisions for collection and use of participant data and biological specimens {26b}

Participants can optionally consent to (1) the storage and usage of their identifiable videos for future research related to mental health disorders and (2) the submission of their deidentified data to the National Institute of Mental Health Database (NDA) and the National Institutes of Health (NIH).

## Interventions

### Explanation for the choice of comparators {6b}

rTMS protocols involving both “inhibitory” pSMA stimulation and “excitatory” left dlPFC stimulation have demonstrated safety and clinical benefits in adult OCD, [25] but these have not yet been examined in the same study or tested in youth with OCD. We selected TBS stimulation protocols because these have been shown to be safe and tolerable in children, involve a brief duration of stimulation (2–3 min), and can be applied with lower stimulation intensity than conventional rTMS protocols [29]. Sham stimulation acts as a placebo comparison for active TMS conditions.

## Intervention description {11a}

**Fig. 2:**
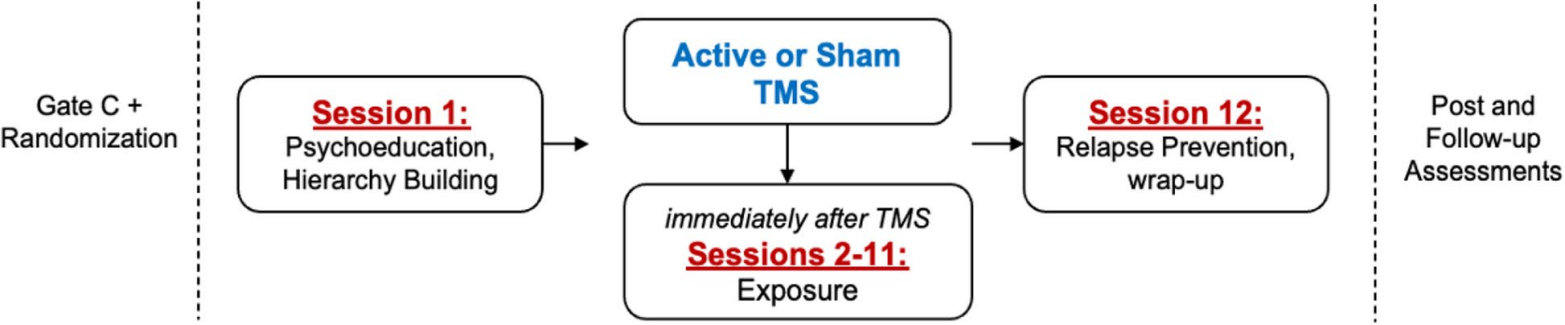
Flow chart of treatment sessions

Participants will receive 12 treatment sessions, delivered daily on consecutive weekdays. The first and twelfth sessions will be ERP orientation and relapse prevention sessions, respectively. Treatment sessions 2–11 will involve TMS immediately followed by ERP (Fig. 2). The number and timing of sessions was designed to ensure delivery of a full course of efficacious ERP [3, 4] alongside daily TMS, which is linked to long-lasting neural and behavioral change [30].

### TMS protocol

Both sites will use the Magstim Super Rapid2 stimulator (Magstim Company Ltd, UK) with a Magstim air-cooled 70-mm figure-eight coil for motor threshold determination and active TMS delivery. The Magstim sham air-cooled coil will deliver sham stimulation. This coil appears and sounds identical to an active coil but delivers a minimal portion of the magnetic field (< 3%). Stimulation intensity for all treatment sessions will be based on the resting motor threshold (RMT) obtained at the second treatment session, prior to the first TMS intervention session. Single-pulse TMS will be administered to the contralateral hand area of primary motor cortex to obtain the RMT, defined as the minimum intensity setting that elicits a threshold electromyography (EMG) response (≥ 50 mV in peak-to-peak amplitude) in a target muscle (abductor pollicis brevis) at rest in 5/10 trials.

The TMS coil placement will be individually determined using an automated neuroimaging analysis pipeline modeled after Lynch et al [31]. This pipeline integrates information about brain anatomy (structural MRI images), resting-state functional connectivity network maps (fMRI), and the modeled TMS-induced electric field (finite element method within the SimNIBS toolbox [32]) to output the coil location (*x, y, z* coordinates) for left dlPFC and pSMA targets. Coil orientation will be held constant for ease of administration. Coil placement will be guided by a stereotactic neuronavigation system (BrainSight 2.3.5, Rogue Research, Montreal, Quebec, Canada) using individual anatomical MRI data.

TMS parameters are as follows: (1) cTBS protocol: bursts of 3 pulses at 30 Hz repeated every 200 ms (5 Hz burst frequency), single uninterrupted 40 s train, 600 total pulses at 90% resting motor threshold (40 s duration); (2) iTBS protocol: bursts of 3 pulses at 30 Hz repeated every 200 ms for 2 s (1 train), trains repeat every 10 s (8 s inter-burst interval between trains), 600 total pulses at 70% resting motor threshold (190 s duration); (3) sham protocol: to enhance masking, half of sham participants will be exposed to the cTBS sequence and half will be exposed to the iTBS sequence.

### ERP protocol

ERP delivery will follow a principle-based treatment manual adapted from the POTS trials [33, 34] and tested in intensive (daily) formats [35]. The manual includes developmental adaptations to tailor delivery for different age groups. This manual was designed to incorporate exposure into each of sessions 2–11 and to maximize therapist delivery factors contributing to successful response prevention during exposures [6, 36]. All exposures will be conducted in-office to ensure that they can be captured on video for Exposure Process Coding System (EPCS) coding [6, 36] while maintaining privacy.

### Criteria for discontinuing or modifying allocated interventions {11b}

If needed, TMS stimulation intensity may be adjusted following review by a study physician. Participants may be withdrawn by investigators for any of the following reasons: (1) significantly deteriorating clinical course (e.g., acute suicidality); (2) significant adverse reaction to TMS; (3) serious physical illness; (4) significantly interfering non-compliance with study procedures; or (5) the participant no longer meets inclusion criteria. Participants will be free to decide to withdraw at any time for any reason. Study withdrawals will be documented, and we will make every attempt to conduct the remaining assessments and provide referrals when needed.

### Strategies to improve adherence to interventions {11c}

Coil placement during TMS administration will be continually monitored using BrainSight (BrainSight 2.3.5, Rogue Research, Montreal, Quebec, Canada). All ERP sessions will be video recorded and 100% will be coded using the Exposure Process Coding System (6) to measure compulsions and therapist and patient adherence. Participant compliance with ERP assignments and in-session tasks will be tracked by the ERP therapist at each session.

### Relevant concomitant care permitted or prohibited during the trial {11d}

To increase external validity of findings, we will include participants taking psychotropic medications that meet the study stability criterion and individuals who previously received OCD-specific therapy if they meet the OCD severity criterion. Concomitant psychotherapy is allowable if it is not focused on OCD. Participant medications and treatments will be checked daily prior to each treatment session. Any change will prompt principal investigator and/or physician review. Prior medications and psychotherapy will be recorded.

### Provisions for post-trial care {30}

The study will not include specific provisions for ancillary or post-trial care. Relevant treatment recommendations and referrals will be provided if participants/caregivers ask or if licensed clinical staff think the participant would benefit from additional clinical care.

### Outcomes {12}

Study assessment measures are listed in Table 1.

**Table 1:** Study assessment measures and procedures

Assessment/Procedure	Purpose	Reporter	Administration Time						
			**Gate A**	**Gate B**	**Gate C**	**Sessions**	**Mid-Tx**	**Post-Tx**	**1-Month**
Phone screen	Initial Eligibility	Pa, Pt	x						
TMS/MRI-Safety Screen [51]	TMS and MRI safety	Pa, Pt	x						
Medical records	Current medications, relevant documents	SS	x						
Informed consent	Informed consent	–		x					
Concomitant treatment	Medication and therapies tracking	SS		x	x	x		x	x
Demographics	Sample characteristics	Pa, Pt		x					
Tanner Stage	Development	Pt		x					
DIAMOND-Kid [52]	DSM-V diagnoses	IE		x					
Children’s Yale-Brown Obsessive Compulsive Scale (C/YBOCS) [53]	OCD symptom severity, subtype	IE		x	x		x	x	x
Clinical Global Impressions (CGI) [54]	Global severity/improvement	IE		x	x		x	x	x
Ask Suicide-Screening Questions (ASQ) [55]	Suicidal ideation	Pt		x	x	x		x	x
Functional Magnetic Resonance Imaging (fMRI)	TMS targeting, RSFC outcome	–			x			x	
Neurocognitive tasks	Cognitive function	Pt			x		x	x	
Masking Questionnaire	TMS masking adequacy	Pa, Pt, SS						x	
TMS Adverse Events Questionnaire (AEQ) [56]	Side Effects/Adverse Events	Pt, SS				x		x	x
Client Satisfaction Questionnaire (CSQ-8) [57]	Treatment Satisfaction	Pa, Pt						x	x
Exposure Process Coding System (EPCS)* [28]	Compulsions; ERP Fidelity	SS				x			
Child Sheehan Disability Scale (CSDS) [58]	Impairment	Pa, Pt			x			x	x
Revised Children’s Anxiety and Depression Scale (RCADS) [59]	Anxiety/Mood	Pa, Pt			x			x	x
Behavior Rating Inventory of Executive Function (BRIEF) [60]	Executive Functioning	Pa, Pt			x			x	x
PROMIS Sleep Disturbance short form [61]	Sleep	Pt			x			x	x
Generalized Anxiety Disorder (GAD-7) [62]	Anxiety	Pa, Pt			x			x	
Patient Health Questionnaire (PHQ-9) [63]	Health	Pa, Pt			x			x	
WHO Disability Assessment Schedule (WHODAS-2) [64]	Functioning	Pa, Pt			x			x	
Menstrual Cycle	Cycle phase	Pt		x	x			x	x

*Pa* parent, *Pt* participant, *SS* study staff, *IE* independent evaluator

#### Phase 1

Primary outcomes focus on neural change and will be indexed by within-subject (pre- to post-treatment) and between-subject (iTBS vs. sham; cTBS vs. sham) change in fMRI-measured RSFC between the targeted network (pSMA-DLS for cTBS arm, dlPFC-DMS for iTBS arm). We will also examine several other outcomes. First, we will evaluate between-subject differences in compulsions during ERP (rate per session as observed by independent raters using EPCS). We will assess safety at baseline, post-treatment, daily treatment sessions, and 1-month follow-ups using the staff-administered Adverse Events Questionnaire (TMS AEQ), aggregated as the number of treatment-related adverse events and tolerability ratings of side effects. We will assess feasibility at post-treatment, based on treatment completion rate (percent of participants completing ≥ 80% of treatment sessions).

#### Phase 2

The primary outcomes are neural change (RSFC in the targeted circuit as in Phase 1) and change in compulsion rate (rate per session as in Phase 1). These will be indexed by within-subject (pre- to post-treatment) and between-subject (active vs. sham) change. We will also examine whether changes in neural and behavioral outcomes mediate change in OCD symptom severity (percent change in C/YBOCS total score from pre- to post-treatment). Secondary analyses will examine (1) the relationship between pre-treatment clinical measures and OCD symptom change, and (2) subgroup effects in primary outcomes, and (3) durability of change in primary outcomes through 1-month follow-up.

### Participant timeline {13}

Participant activities are depicted in Fig. 1. Participants who meet initial eligibility criteria on the phone screen (Gate A) will be scheduled for the clinical interview (Gate B), at which time the consent/assent and Gate B measures will be completed and final eligibility determined. The baseline assessment (Gate C), which involves the pre-treatment MRI scan, will be completed within 1 calendar month of Gate B. The daily treatment sessions will begin within 5 calendar days of completing Gate C (to allow for running and checking the TMS targeting pipeline). Participants will complete the 12 TMS + ERP treatment sessions (Fig. 2) within 15 business days. The mid-treatment assessment will occur between sessions 6 and 7. The post-treatment assessment will be completed within the 10 calendar days following the last treatment session, with a targeted window of 1–3 business days. The post-treatment MRI scan will occur at least 24 h after the last TMS session to avoid capturing the acute aftereffect of TMS. The 1-month assessment will be completed 4–6 weeks after the last treatment session.

### Sample size {14}

Power analyses for our sample sizes (phase 1 = 60, phase 2 = 60), assuming an attrition rate of 10%, (phase 1 = 54, phase 2 = 54) were calculated. In Phase 1, we will have 80% power to detect medium-to-large effects for within- and between-subject changes in RSFC (Cohen’s d range 0.70 to 0.96) and for group differences in compulsion rate (η2 = 0.23). In Phase 2, we will have 80% power to detect medium-to-large effects for tests of group differences in RSFC and compulsion rate (Cohen’s *d* = 0.78) and for tests of RSFC and compulsion rate as mediators of OCD symptom change (η2 range = 0.16 to 0.20).

### Recruitment {15}

Study information will be distributed to clinicians within the care systems of each site and across community practices in each region, focusing on those most likely to encounter individuals with OCD (i.e., psychiatrists, psychologists, primary care physicians). Advertising will occur via physical flyers posted in public spaces and digital flyers shared via lab social media accounts, departmental websites, and OCD-related patient support organizations. There will be separate recruitment for Phase 1 and Phase 2 of the randomized controlled trial (RCT).

### Assignment of interventions: allocation

#### Sequence generation {16a}

Block randomization, stratified on site (Brown or UMN) and baseline OCD severity (C/YBOCS total < 32 vs. > = 32), will be performed using the blockrand package in R [37].

#### Concealment mechanism {16b}

Conditions are concealed within a digital randomization key that is only accessible to unmasked staff.

#### Implementation {16c}

Stratification information will be sent to the study statistician via Research Electronic Data Capture (REDCap). The study statistician will complete the randomization, the TMS supervisor will verify the randomization, and then the TMS operators will be informed of protocol type (cTBS or iTBS) and coil (active vs. sham coils are concealed with coded letters or colors). A separate active coil will be used for RMT determination to enhance masking.

### Assignment of interventions: Blinding

#### Who will be blinded {17a}

People who will be masked to TMS status (active vs. sham) are participants, caregivers (if applicable), and study staff involved in the clinical assessments (independent evaluators, IEs), coding the ERP videos, delivering ERP therapy, delivering TMS, and collecting the MRI data. The staff involved in the clinical assessments and coding the ERP videos will not be present for a given participant’s TMS + ERP sessions to ensure masking to overall treatment progress. In Phase I, some people will necessarily be aware of cTBS vs. iTBS allocation (TMS operator, participants, and caregivers present for TMS) but will remain masked to active vs. sham status. In phase 2 these individuals will be fully masked. After treatment, forms assessing masking adequacy will be given to masked staff who conduct TMS, ERP, and assessment visits. Unmasked staff will be the study statistician and the TMS site supervisor.

### Procedure for unblinding if needed {17b}

Intentional unmasking of TMS status (active vs. sham) will be allowable if deemed necessary for participant safety, to address a TMS device issue, or another unforeseen situation in which status knowledge is essential for study conduct. Only individuals deemed most critical for addressing the situation will be unmasked. IEs will not be intentionally unmasked.

## Data collection and management

### Plans for assessment and collection of outcomes {18a}

Study measures, reporters, and administration timing are listed in Table 1. Assessments will be conducted by IEs via HIPAA-compliant video conferencing. IEs will be otherwise uninvolved with the study and will be masked to group assignment, treatment progress, and EPCS coding. IEs will be bachelor’s degree-level research staff trained to criterion to administer all IE measures; they will meet weekly for criterion maintenance and be monitored to prevent drift.

### Measure procedures

#### Exposure Process Coding System (EPCS)

EPCS [6, 36] is an observer-rated, microanalytic, time-stamped coding system that uses Noldus Observer software to capture in-session behaviors as they occur on video. EPCS measures the frequency and duration of youth compulsions during exposure tasks, along with codes measuring therapist behaviors (which serve as an index of treatment fidelity) and youth distress levels (youth-reported and observer-rated; which can index exposure learning). Bachelor’s degree-level study coders will code 80% of video-taped session data. Coders will be trained and monitored to prevent drift. Training will include background reading, group discussions, practice coding with feedback, and practice coding to criterion. Raters must attain reliability on practice videos (*k* > 0.80) before coding study videos. If coder drift is detected, re-training will occur and remaining study videos will not be coded until the reliability criterion is re-established. Ten percent of sessions will be double-coded to assess reliability.

### MRI and fMRI data acquisition

Siemens Prisma 3T scanners located at the Brown University MRI Facility or University of Minnesota Center for Magnetic Resonance Research will be used for image acquisition. Total MRI time will be ~ 1 h for each visit. To minimize motion artifact, we will immobilize the participant’s head with foam padding. We will use Framewise Integrated Real-Time MRI Monitoring (FIRMM) during all functional scans to monitor subject motion in real-time and track the amount of usable data, aiming for ≥ 10 min of data with FD < 0.2 mm, with respiration filter [38]. As needed, we will run additional scan(s) to meet the usability criterion. We will acquire scans using the ABCD pulse sequences, using a multi-echo, multiband BOLD acquisition.

1. A high-resolution volumetric navigator for prospective motion correction [39].
2. Structural scans: whole brain 3D T1-weighted (176 slices, 1 mm isotropic, TE = 2.88 ms, TR = 2500 ms, flip angle = 8°) and whole brain 3D T2-weighted images (176 slices, 1 mm isotropic, TE = 564 ms, TR = 3200 ms), during which participants can watch a movie.
3. Resting state fMRI: whole-brain BOLD-sensitive EPI sequence, multi-echo, multi-band scans (TR = 1761 ms, TE = 14.20/38.94/63.66/88.40 ms, flip angle = 68°, 72 oblique axial slices, 2.0 mm isotropic resolution, Multi-band factor = 6, GRAPPA = 2, 412 volumes, 12 min each). Two 12-min runs will be collected during each visit. Participants will be asked to rest quietly, try to stay awake, and watch a fixation cross.

### MRI and fMRI pre-processing

Functional data will first be denoised with NORDIC [40] and then the multi-echo data will be combined using the “tedana” software package to maximize signal to noise [41]*.* The ABCD-BIDS pre-processing pipelines will be applied to the structural T1 and T2 data as well as the denoised resting state data [42]. Functional time series data will be further preprocessed to reduce artifacts, and subjected to stringent frame censoring and additional nuisance regression to account for potential movement confounds [43]. For analyses, we will extract resting-state BOLD timeseries from an established set of 300 well characterized regions of interest (ROIs) across the whole brain (239 cortical, 34 subcortical, 27 cerebellar) [44] and correlate the timeseries region-by-region to generate brain-wide correlation matrices. pSMA and dlPFC seeds will be the region of interest (ROI). The DLS seed will be ROIs in putamen and the DMS seed will be ROIs in the caudate tail.

### Plans to promote participant retention and complete follow-up {18b}

All study visits will be scheduled in advance, and staff will work with adult participants and parents to problem-solve potential barriers to attending study visits. Family-friendly visit times will be offered as needed. We have also budgeted funds to assist families with transportation costs if that is a barrier. We will implement the Adjunctive Services and Attrition Prevention (ASAP) plan to minimize participant attrition and effectively manage clinical crises that do not meet criteria for study withdrawal, which may occur in RCTs involving a clinical sample. An “ASAP” event is any situation that requires a brief intervention by study staff beyond what is afforded by the assigned treatment arm. Each participant will be allowed one ASAP session during the acute treatment phase.

### Data management {19}

The web-based REDCap system will be used for secure data entry and storage. All entered data will be double-checked by a second staff member, and range checks and missing data flags will be automated. Imaging data will be deidentified and stored on secure servers. A HIPAA-compliant secure storage platform, Box, will be used to store video recordings and data back-ups.

### Confidentiality {27}

Participant information and data will only be accessible to study personnel who (1) have up-to-date human subjects training and certification through the Collaborative Institutional Training Initiative (CITI) curriculum, (2) complete HIPAA and Protected Health Information (PHI) training through their respective institutions, and (3) maintain up-to-date certification on research subject confidentiality and privacy. Data will be labeled using subject ID numbers stored separately from identifying information.

### Plans for collection, laboratory evaluation and storage of biological specimens for genetic or molecular analysis in this trial/future use {33}

Not applicable as biological specimens will not be collected.

## Statistical methods

### Statistical methods for primary and secondary outcomes {20a}

#### Phase 1

##### Primary outcome (connectivity in targeted circuit)

Seed-based analyses [44] will test RSFC between a priori identified ROIs (as described above) and use a false discovery rate (FDR) correction for multiple comparisons threshold of *p* < 0.05. ROI correlation matrices will be converted to individual *Z*-scores using Fisher’s transformation, yielding indices representing RSFC in each targeted network. To test if active rTMS has a within-subject effect on RSFC, we will conduct paired *t*-tests for the change in RSFC for each active rTMS group within the targeted network (pSMA-DLS for cTBS arm, dlPFC-DMS for iTBS arm). We will conduct two separate two-sample *t*-tests for the group effect of rTMS: one for iTBS (dlPFC) vs sham and one for cTBS (pSMA) vs sham.

##### Secondary outcome (compulsion duration)

EPCS-rated duration of observed compulsions (in seconds, summed across all sessions) will quantify compulsive behavior. We will fit two linear regression models with compulsion duration as the dependent variable to establish if there exist differences in how iTBS, cTBS, and sham conditions affect compulsion duration. Group indicators for these conditions (active vs sham) will be the primary model predictors. Given that the overall exposure duration naturally varies across patients and sessions, [36] relates to ERP outcomes, and can influence compulsion duration (i.e. longer exposures have more opportunity for compulsions), total exposure duration (in seconds) will be a covariate in the model.

### Other outcomes

We will explore group differences on secondary measures of clinical functioning listed in Table 1.

#### Phase 2

##### Primary outcome (connectivity in targeted circuit)

Following procedures outlined for this outcome in Phase 1, a two-sample *t*-test will assess the difference in RSFC in the targeted circuit pre- and post-treatment for active rTMS vs sham. A linear regression model will also be used with the outcome of interest being the percent change in OCD symptom severity (C/YBOCS) pre- and post-treatment. The primary predictor will be a group indicator for sham vs active TMS. Differences pre- and post-treatment in RSFC and its interaction with the group indicator will be included as potential mediators for the dependent variable.

##### Primary outcome (compulsion duration)

Following procedures outlined for this outcome in Phase 1, a linear regression model will assess whether compulsion duration differs by treatment group. A linear regression model will also be used with the outcome of interest being the percent change in OCD symptom severity (C/YBOCS) pre- to post-treatment. The primary predictor will be a group indicator for sham vs active rTMS. Compulsion duration and its interaction with the group indicator will be included as potential mediators for the dependent variable. Total exposure duration will be a covariate in both analyses.

##### Other outcomes

We will explore the durability of treatment-related change in OCD symptoms by testing group differences in clinical change on the C/YBOCS at post-treatment and 1-month follow-up. Group differences on secondary measures of clinical functioning listed in Table 1 will be explored.

### Interim analyses {21b}

This trial has no planned interim analyses. The Data Safety Monitoring Board (DSMB) can request interim analyses to determine whether there is any change to the anticipated benefit-to-risk ratio of study participation.

### Methods for additional analyses (e.g. subgroup analyses) {20b}

In both study phases, we will conduct exploratory analyses to investigate baseline participant characteristics as predictors of change in RSFC and compulsion duration across treatment arms. OCD is a highly heterogeneous disorder typically accompanied by one or more comorbidities, and for which subgroup differences may be particularly relevant. Specifically, we will explore the relevance of the following baseline characteristics: comorbidity (yes/no: mood, anxiety, ASD, ADHD, tics), OCD symptom subtype (harm avoidance vs. incompleteness), presence of mental compulsions (yes/no), assigned sex at birth, and Tanner stage (stages 3–4 will be coded post pubertal).

### Methods in analysis to handle protocol non-adherence and any statistical methods to handle missing data {20c}

We will implement multiple imputation methods for any missing data prior to our analyses, assuming a missing at random missingness mechanism using Multiple Imputation by Chained Equations (MICE) in the *mice* package in R. MICE allows flexibility in predictor variable types (binary, continuous, multinomial, etc.). All randomized participants will be used in analyses.

### Plans to give access to the full protocol, participant-level data and statistical code {31c}

De-identified participant data will be submitted to the National Institute of Mental Health Data Archive (NDA, Collection #C4911) if participants opt in during the consent process. The code for statistical analyses and the MRI scanning protocol can be made available upon request.

## Oversight and monitoring

### Composition of the coordinating center and trial steering committee {5d}

This multi-site study is organized into the following “teams”: oversight, data coordination, data analysis, imaging, TMS, ERP, independent evaluator (IE) assessments, and EPCS coding. The oversight team, led jointly by the PIs, will provide global oversight of study conduct, procedural and scientific integrity, regulatory management, data collection, quality assurance, data analysis, and dissemination of findings. The cross-site implementation teams will each be led by a doctoral-level co-investigator, with each site taking the lead on teams that best leverage site expertise (TMS, imaging, and data coordination by UMN; ERP, IE assessments, and EPCS coding by Brown). Each team will meet weekly or bi-weekly and work with the oversight team to develop and monitor Standard Operating Procedures (SOPs).

#### Composition of the data monitoring committee, its role and reporting structure {21a}

An independent DSMB is in place for this study. Members have expertise in biostatistics, neurodevelopmental disorders, and clinical care of youth with OCD. DSMB members are not involved in this project and are not collaborators on any other of the investigators’ projects nor in their employ. DSMB policies and responsibilities are outlined in a charter created prior to the start of data collection. Annual DSMB meetings will be held to monitor study conduct, safety data, and participant safeguards. De-identified reports to the DSMB will include information about study progress, participant accrual, adverse event data, protocol deviations, and external factors or information relevant to the study and its risk–benefit ratio. The DSMB has the authority to vote on study continuation and make recommendations about study procedures, and they can be contacted by regulatory authorities or similar entities seeking advice about any potential concerns.

### Adverse event reporting and harms {22}

The Adverse Events Questionnaire (AEQ) will be administered at every TMS study visit, and staff will conduct general inquiries regarding health status between visits. For each endorsed AE, data will indicate severity, relatedness to TMS, relatedness to ERP, and actions taken. All adverse events will be reviewed by the PIs. AE reporting procedures are in accordance with policies of the IRB, FDA, NIH, and site institutions. Annual IRB progress reports will include (1) adverse events deemed expected or unrelated to the study and (2) protocol deviations that do not affect the scientific soundness of the research plans or the rights, safety, or welfare of the human subjects. Promptly reportable events include (1) unanticipated serious adverse events, (2) unanticipated problems involving risks to subjects or others related to research participation, (3) information reports indicating new or increased risk with the investigational device, and (4) protocol deviations that increased risk of harm.

The study will be stopped should any of the following occur: (1) two participants experience a generalized-onset seizure, (2) two participants experience a focal-onset seizure with impaired awareness, (3) two participants experience a seizure for which anticonvulsant medication is administered, (4) one participant experiences status epilepticus or is hospitalized due to a seizure(s), (5) two or more participants attempt suicide or one participant completes suicide, (6) two or more device-related serious adverse events (SAEs) in one or more participants; OR a single device-related SAE in five or more participants, or (7) two or more participants show a decrease in working memory at any time during the study. The likelihood of a seizure occurring in this trial is extremely low given the TMS parameters being used and the design of inclusion/exclusion criteria to exclude participants with elevated risk [45].

### Frequency and plans for auditing trial conduct {23}

The NIMH Clinical Research Education, Support, and Training Program (CREST) will provide regular monitoring and evaluation. Monitoring visits will occur during study initiation, yearly after that, and after completion of data collection. The monitoring schedule may be adjusted depending on factors like accrual rate, protocol deviations, or DSMB recommendation.

### Plans for communicating important protocol amendments to relevant parties (e.g., trial participants, ethical committees) {25}

IRB approval will be obtained prior to any protocol changes. Investigators will follow FDA rules pertaining to investigational devices with significant risk. Under these rules, investigators will submit annual reports to the FDA and will obtain FDA approval before making significant changes to the protocol (i.e., changes that impact risk and/or scientific integrity). Significant protocol changes will also be posted to ClinicalTrials.gov and reported to the DSMB. If there are changes to the informed consent, participants who have not completed the study will be re-consented/assented.

#### Dissemination plans {31a}

Results will be submitted to ClinicalTrials.gov via the Protocol Registration and Results System Information website. A lay summary of results will be sent to participants once results are published.

## Discussion

The NExT trial will test, for the first time, the combination of ERP and TMS in pediatric OCD and the resulting effects on neural, behavioral, and clinical functioning. The phased design will enable us to initially compare safety, tolerability, and different TMS dosing strategies to understand intervention effects on neural and behavioral targets prior to starting a second phase powered to establish the relationship between neural/behavioral target engagement and OCD symptoms. Results will inform TMS therapy augmentation strategies; improve our understanding of neural mechanisms underlying ERP, TMS, and pediatric OCD; and, if safe and efficacious, potentially lead to a novel treatment option for youth with OCD.

This study is aligned with other recent efforts to test the potential benefits of TMS-based treatment in younger patients, particularly because TMS has demonstrated safety, tolerability, and feasibility in children and adolescents and is associated with fewer side effects than commonly prescribed medications [46]. Testing specific TMS protocols head-to-head in children, rather than simply testing the adult TMS OCD protocol, is important. Childhood-onset OCD has been shown to differ from adult OCD in terms of symptom topography, genetic influences, gender distribution, comorbidity profile, neural circuit function, and degree of insight, e.g., [47].

We elected to focus specifically on adolescence for several reasons. First, the clinical need for ERP augmentation typically arises later in development after current first-line treatments for pediatric OCD have been tried. Second, fear extinction learning can be less robust in adolescents vs. children and adults [48]. Finally, adolescence is a critical period for the development of higher-order cognitive abilities, a process driven by heightened neuroplasticity that can be strongly affected by experience and learning [49]. Because of this, interventions that directly modulate neural circuits alongside focused behavioral engagement may be well-poised to capitalize on the neural milieu of adolescence. The extent to which TMS-based interventions are effective or need to be adapted to particular developmental stages should continue to be investigated. These adaptations should include considerations about neurocognitive development, measures to enhance comfort/tolerability, and practicalities such as intervention schedule and time demands on children and families.

This trial focuses on pairing ERP with TMS rather than on TMS monotherapy. Prior adult studies focused exclusively on using TMS after “symptom provocation” to alter the circuitry underlying OCD pathology [50]. Our aim is to specifically target the circuitry supporting response prevention, a “therapy critical” behavior thought to be essential to the success of ERP (i.e., fear extinction). Augmentation strategies that improve mechanism engagement in existing treatments may be most likely to enhance treatment response vs. those that have merely an additive effect. We also recognize that complete demonstration of a synergistic effect of ERP + TMS would necessitate the inclusion of TMS only treatment arms. However, we did not include TMS only or TMS + “therapy placebo” arms because (1) our central goal is centered on an augmentation question designed to improve response to the existing first-line treatment for OCD, and (2) we felt it unethical to deliver “therapy placebo” since ERP has already demonstrated superiority. Future research testing TMS monotherapy may be most clinically relevant if focused on those who are unable to fully participate in ERP (e.g., due to cognitive/language disorders, refusal or inability to engage in exposure tasks) or on questions of treatment sequencing.

In terms of including youth with comorbidities and those taking medication, we prioritized generalizability to ensure representation of patients who most often need ERP. As common OCD comorbidities reflect shared underlying deficits in habit or goal-directed systems (e.g., tics, ADHD), exclusion of these youth may inadvertently omit some who could benefit most from TMS augmented ERP. Design elements, such as block randomization, will account for this heterogeneity, and we will also conduct subgroup analyses to the extent possible in order to understand how these factors may impact response.

Finally, a few other design choices are worth noting. In terms of TMS stimulation targets, we considered the orbitofrontal cortex (OFC) because it is also implicated in habit and goal-directed systems in OCD. While a promising target, adults receiving TBS to OFC report a high level of pain during stimulation [24]. pSMA and dlPFC targets are more tolerable because stimulation induces minimal discomfort and facial muscle movement. This is particularly critical for a first test in adolescents, who have higher motor thresholds relative to adults and for whom careful attention to comfort and safety is warranted.

### Trial status

This report is based on protocol version 1 (September 20, 2023). Recruitment began February 2024. Recruitment for phase 1 is expected to be complete by August 2025. If phase 1 milestones are met, phase 2 is anticipated to begin shortly after approval and have a duration of 3 years.

## Data Availability

Data will be submitted to the NIMH NDA every 6 months and held privately until shared with the research community at the time of paper publication acceptance and/or the end of the award period.

## Abbreviations

AEQ: Adverse Events Questionnaire
ASAP: Adjunctive Services and Attrition Prevention
CITI: Collaborative Institutional Training Initiative
CMRR: Center for Magnetic Resonance Research
CNBD: Center for Neurobehavioral Development
CREST: Clinical Research Education, Support, and Training Program
cTBS: Continuous theta burst stimulation
C/YBOCS: Child/adult Yale-Brown Obsessive Compulsive Scale
dlPFC: Dorsolateral prefrontal cortex
DLS: Dorsolateral striatum, aka putamen
DMS: Dorsomedial striatum, aka caudate
DSMB: Data Safety Monitoring Board
EMG: Electromyography
EPCS: Exposure Process Coding System
ERP: Exposure with Response Prevention
FDA: United States Food and Drug Administration
FDR: False discovery rate
FIRMM: Framewise integrated real-time MRI monitoring
fMRI/MRI: Functional/magnetic resonance imaging
HIPAA: Health Insurance Portability and Accountability Act
IE: Independent evaluator
IRB: Institutional Review Board
iTBS: Intermittent theta burst stimulation
MICE: Multiple imputation by chained equations
NDA: National Institute of Mental Health Database
NIH: National Institute of Health
NNL: Non-invasive Neuromodulation Laboratory
OCD: Obsessive-Compulsive Disorder
OFC: Orbitofrontal cortex
PARC: Pediatric Anxiety Research Center
PHI: Protected health information
PI: Principal Investigator
p/SMA: Pre/supplementary motor area
RCT: Randomized controlled trial
REDCap: Research Electronic Data Capture
RMT: Resting motor threshold
ROI: Region of interest
RSFC: Resting state functional connectivity
rTMS/TMS: Repetitive/transcranial magnetic stimulation
SAE: Serious adverse event
SRI: Serotonin reuptake inhibitor
